# Innovations in Imaging: ^18^F-Fluorodeoxyglucose PET/CT for Assessment of Cardiovascular Infection and Inflammation

**DOI:** 10.1007/s11886-024-02137-z

**Published:** 2024-09-24

**Authors:** Siddharth J Trivedi, Jamieson M Bourque

**Affiliations:** 1 Cardiovascular Division (Department of Medicine), Department of Radiology, Brigham and Women’s Hospital, Harvard Medical School, Boston, MA USA; 2 Division of Cardiovascular Medicine, Cardiac Imaging Center, Departments of Medicine and Radiology, University of Virginia, 1215 Lee Street, PO Box 800158, Charlottesville, VA 22908 USA

**Keywords:** ^18^F-FDG, PET/CT, Cardiovascular Infection, Inflammation, Cardiac Imaging, Nuclear Cardiology

## Abstract

**Purpose of review:**

^18^F-Fluorodeoxyglucose positron emission tomography (PET) combined with computed tomography (CT), referred to as ^18^F-FDG PET/CT, plays a significant role in the diagnosis and management of patients with systemic infectious and inflammatory conditions. This review provides an overview of ^18^F-FDG PET/CT in systemic infectious and inflammatory conditions, including infective endocarditis (IE), cardiac implantable electrical device (CIED)/left ventricular assist device (LVAD) infection, sarcoidosis, and large-vessel vasculitis (LVV).

**Recent Findings:**

This review highlights the past and present literature in the increasing role of ^18^F-FDG PET/CT in cardiovascular inflammation and infection, including diagnostic and prognostic findings.

**Summary:**

They key aspects of this paper are to highlight the importance of ^18^F-FDG PET/CT in cardiovascular infection and inflammation, and to provide illustrations of how it can contribute to patient diagnosis and management.

## Introduction

Positron emission tomography myocardial perfusion imaging (PET MPI) is well-established as a superior functional imaging modality in the assessment of ischemic heart disease, including obstructive epicardial coronary artery disease (CAD) and coronary microvascular dysfunction (CMD) in patients with ischemia and no obstructive CAD (INOCA). Tracer applications in CAD beyond perfusion have enabled assessment of added domains, including myocardial viability, atherosclerotic plaque activity and extent of cardiac innervation in heart failure [1]. Beyond ischemic heart disease, cardiovascular PET imaging combined with computed tomography (PET/CT) has growing use in systemic diseases with cardiovascular manifestations [1]. The clinical and imaging complexity of these diseases mandate a standardized imaging approach [2]. Novel tracers show exciting potential in infiltrative disease such as ATTR and AL cardiac amyloidosis [3]. However, the broadest growth in use of cardiovascular PET beyond ischemic heart disease has been in the assessment of inflammatory and infectious cardiovascular complications of systemic diseases. This technique uses 2-deoxy-2-[18F]fluoro-D-glucose (^18^F-FDG), which accumulates in cells with metabolic glucose utilization. The high glucose metabolism of white blood cells and other inflammatory cells in infected and inflamed tissue facilitates their imaging with ^18^F-FDG PET/CT [1]. This review focuses on the diagnostic and prognostic impact of ^18^F-FDG PET/CT and management applications in patients with systemic infectious and inflammatory conditions, including infective endocarditis (IE), cardiac implantable electrical device (CIED)/left ventricular assist device (LVAD) infection, sarcoidosis, and large-vessel vasculitis (LVV) (Table 1).

**Table 1:** Utility of ^18^F-FDG PET/CT in cardiovascular infection and inflammation

Pathology			Sensitivity	Specificity	Summary
Cardiac Infection	Infective endocarditis	PVE	86% (7)	84% (7)	Combining ^18^F-FDG PET with CT angiography improves diagnostic performance and helps detect peri-prosthetic and peripheral complications
		NVE	31–36% (7)	98–99% (7)	Emerging use – limited sensitivity due to small vegetations; may have a role in assessing extracardiac infection
		Transcatheter valve infection	83% (34)	-	Increasing utility in TAVI, particularly when combined with CT angiography
	Cardiac implanted electronic device infection		Pocket infection: 96% (38); Lead involvement: 97% (38)	Pocket infection: 97% (38); Lead involvement: 83% (38)	Utility is superior for pocket infection compared to lead involvement
	LVAD infection		95% (50)	91% (50)	Promising role of ^18^F-FDG PET/CT as many components of the LVAD are not assessed by echocardiography
Cardiovascular Inflammation	Cardiac sarcoidosis		84% (62)	83% (62)	Quantification of inflammation may provide added value for diagnosis and monitoring of disease progression and response to therapy
	Large vessel vasculitis		76% (83)	93% (83)	Role in patients with symptoms of large vessel vasculitis or fever/inflammation of unknown origin

Abbreviations: ^18^F-FDG, ^18^F-Fluorodeoxyglucose; CT, computed tomography; LVAD, left ventricular assist device; NVE, native valve endocarditis; PET, positron emission tomography; PVE, prosthetic valve endocarditis; TAVI, transcatheter-aortic valve implantation

## Cardiac Infection

The primary and initial imaging tool for cardiovascular infection remains transthoracic and transesophageal echocardiography (TTE and TEE respectively). These modalities have ubiquitous availability, low-cost, and provide high-yield evaluation of structure and function. However, poor imaging quality can impair identification of small infectious processes and differentiation of infectious and non-infectious masses [4]. TTE provides limited assessment of extracardiac structures and does not interrogate remote infectious involvement, including septic emboli and device pockets/hardware. Finally, TEE is an invasive procedure with risks, particularly in the elderly population [5]. Advanced imaging with ^18^F-FDG PET/CT can address many of these limitations and has an important supplementary role in many patients with suspected or known native and prosthetic valve infective endocarditis (IE), and CIED/LVAD infection. Recently-released multi-societal expert consensus recommendations clarify the role of ^18^F-FDG PET/CT in the evaluation of cardiovascular infection [6].

### Infective Endocarditis

#### Prosthetic Valve Infection Endocarditis Diagnostic Assessment

**Fig. 1:**
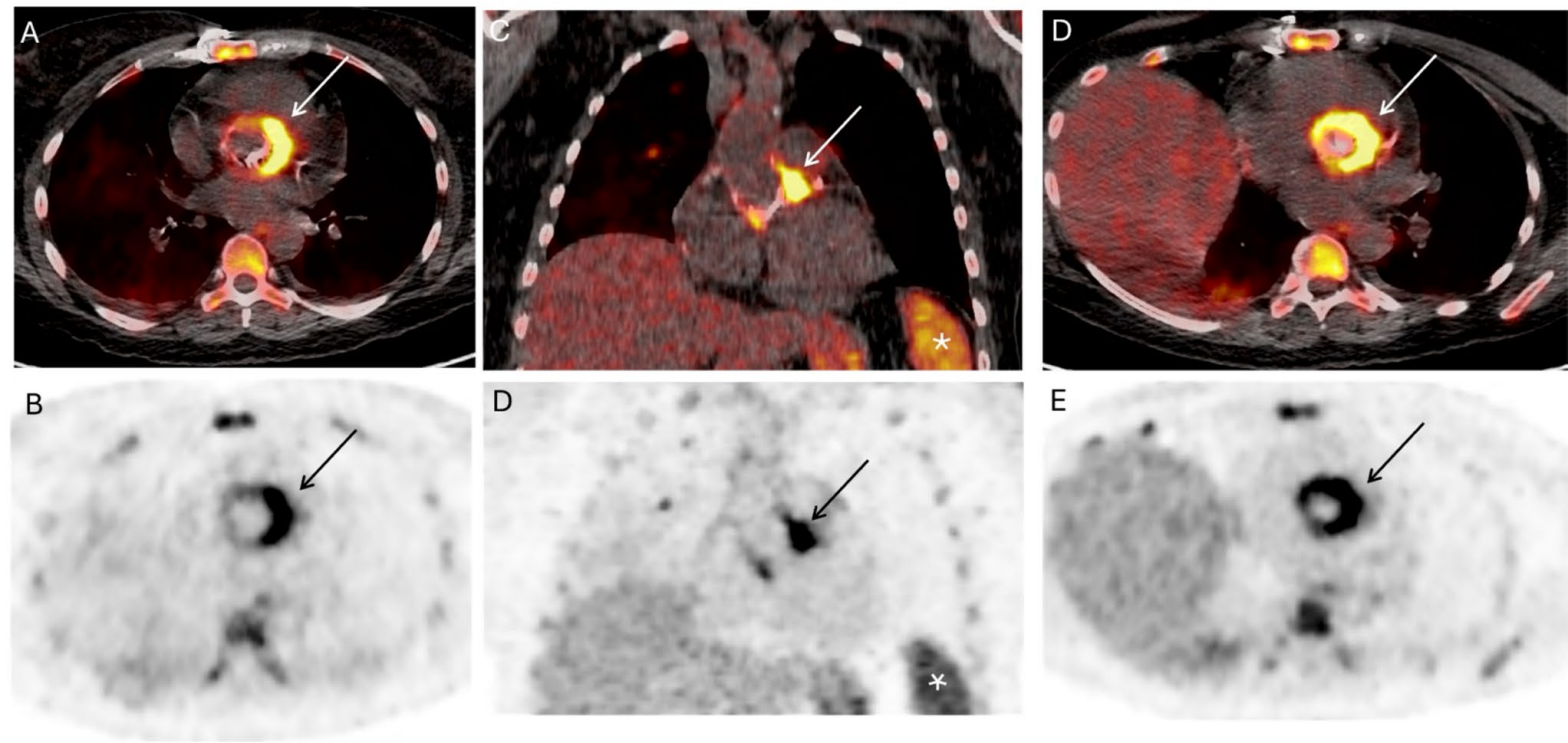
Aortic prosthetic valve infective endocarditis. A 52-year-old female presented with fever and bacteremia in the context of a mechanical aortic valve. A ^18^F-fluorodeoxyglucose (^18^F-FDG) PET/CT imaging study was acquired, obtaining images from the vertex of the skull through the toes, followed by a dedicated cardiac bed acquisition. Fusion and PET images are displayed, images **A** and **B** correspond to the axial plane, images **C** and **D** correspond to the coronal plane, and images **D** and **E** are images oriented to the aortic valve plane. There is intense semi-concentric uptake along the ring of the mechanic aortic valve, higher along the left portion of the valve, concerning for mechanical aortic valve infective endocarditis (*white arrows* on fusion images and *black arrows* on PET images). Additionally, there is increased bone marrow and splenic uptake, most likely related to the underlying inflammatory/infectious systemic process (*white asterisks* on images **C** and **D**)

The literature on the role of ^18^F-FDG PET/CT for the diagnosis of prosthetic valve endocarditis (PVE) is increasing rapidly as the technique offers a number of advantages [7–9] (Fig. 1). The sensitivity of the Duke-Li criteria improves from 70 to 97% when ^18^F-FDG cardiac uptake is used as a major criterion in patients with suspected PVE [10, 11]. This is primarily due to a reduction in the patients with PVE initially labelled as *possible* IE, and appropriately categorizing them as *definite* PVE [11]. A contemporary meta-analysis of 33 patients with PVE across 15 studies concluded pooled sensitivity and specificity of 86% and 84% respectively [7]. The utility of ^18^F-FDG PET/CT is similar for mechanical and biological prosthetic valves [11, 12]. Because of its excellent sensitivity and specificity, the European guidelines recommend ^18^F-FDG PET/CT in patients with suspected PVE and uncertain diagnosis based on the Duke-Li criteria [13]. Furthermore, ^18^F-FDG PET/CT may play a role in diagnosing PVE in patients with slow-growing bacteria and in patients with negative blood cultures [14].

Another imaging modality that can be utilized in cases of diagnostic uncertainty is computed tomography angiography (CTA) [13]. However, although CTA allows detection of vegetations on valve leaflets [15], ^18^F-FDG PET/CT demonstrates early inflammatory signs prior to changes in anatomy [16]. When ^18^F-FDG PET is complemented by CTA, diagnostic utility significantly improves compared to PET with nonenhanced CT [10]. Furthermore, CTA plays an important role in assessing complications of PVE, such as pseudoaneurysms and perivalvular abscesses [16]. The technique also allows for the evaluation of valvular thrombi/vegetation and identification of septic emboli [17]. In addition, ^18^F-FDG PET/CTA assists in determining coronary artery involvement prior to surgical treatment [16].

Patient preparation prior to the examination plays a key role in the diagnostic utility of ^18^F-FDG PET/CT. According to a recent meta-analysis, a prolonged and planned cardiac preparation protocol pre-^18^F-FDG PET/CT resulted in significantly improved performance (sensitivity and specificity of 81.3% and 79.0%, respectively) compared to inconsistencies in specific diet advice to patients pre-imaging (sensitivity 72.3% and specificity 76.2%) [9].

The use of antibiotics prior to imaging influences the diagnostic performance of ^18^F-FDG PET/CT imaging in IE. The intensity of systemic and local inflammation decreases in parallel to the duration of antibiotic therapy, resulting in false-negative ^18^F-FDG PET/CT results [16, 18]. The timing of imaging after prosthetic valve surgery is also important [17, 19]. Indeed, the healing of tissues after surgery generates local inflammation, which can lead to false positive findings. In addition, surgical adhesives and glue induce a sustained inflammatory reaction in the surgical site [20], which may persist several years after prosthetic valve implantation [16, 21]. Consequently, the European Guidelines recommend performing ^18^F-FDG PET/CT after an empirical minimal delay of 1–3 months following surgery [13, 21], a delay that can be reduced to < 3 weeks in case of non-complicated valve surgery and depending on the risk of infection [17].

#### Diagnosis of Native Valve Infective Endocarditis

In comparison to PVE, literature specifically evaluating the role of ^18^F-FDG PET/CT in native valve endocarditis (NVE) is limited [10]. Recent meta-analyses have reported pooled sensitivity of 31–36% for NVE detection and pooled specificity at 98–99% [7, 22]. Reasons for the poor sensitivity of ^18^F-FDG PET/CT in NVE include the relatively low spatial resolution of PET imaging (~ 5 mm) for the detection of the small (< 10 mm) fibrotic valvular vegetations, with sparse inflammatory infiltrate, that are characteristic of NVE [23]. The addition of ^18^F-FDG PET/CT may be useful in patients with NVE to detect peripheral FDG uptake corresponding to septic emboli that are often missed by conventional imaging and are considered as a minor criterion of IE in the modified Duke-Li criteria [13]. Consequently, adding ^18^F-FDG PET/CT in patients with NVE improves the sensitivity of the modified Duke-Li criteria without affecting its high specificity [23, 24]. In addition to reclassifying patients with NVE, it can also result in a change in their therapeutic management (antibiotic or surgical strategy) [24]. ^18^F-FDG PET/CT may be particularly helpful in patients with an inconclusive TEE [13].

#### Assessment of Extracardiac Complications

An important added application of ^18^F-FDG PET/CT in suspected or known cardiovascular infection is the evaluation of extracardiac complications, including septic emboli, mycotic aneurysms, and portal of entry. In both NVE and PVE, ^18^F-FDG PET/CT offers to identify extracardiac infectious locations of PVE, which are then classified as a minor criterion of the modified Duke-Li criteria [13]. In addition, the presence of increased ^18^F-FDG uptake in the spleen and bone marrow in patients with high likelihood of IE has been shown to be an indirect sign of IE [25, 26]. Furthermore, in patients with confirmed IE, ^18^F-FDG PET/CT has high diagnostic value for the early detection of septic emboli [27], including clinically silent emboli, leading to antibiotic therapy initiation, prolongation, or another management decision [28].

Identifying and treating a portal of entry of IE, with the most common infectious portal of entry being cutaneous (notably in patients using intravenous drugs, classically leading to right-sided IE) and digestive (in particular, colic cancer and oral/dental infection) [29], is crucial as this can lead to the recurrence of sepsis and IE, and also allows additional dedicated treatment [30]. Inclusion of the lower limbs in the field of PET acquisitions is useful to detect a classic complication of IE – asymptomatic mycotic aneurysms, septic grafts that can develop on a peripheral vessel and may evolve towards rupture if not identified and treated early [31].

#### Transcatheter Valve Infection

The transcatheter-implanted aortic valve (TAVI) procedure is an increasingly used method of valve replacement, especially in the elderly population [32]. TAVI can be complicated by IE [33], and detection of TAVI-related IE by echocardiography is limited due to metal artifacts. A recent study showed that while ^18^F-FDG PET with nonenhanced CT had a low sensitivity to diagnose TAVI-related IE (58%), adding CTA significantly improved the sensitivity (83.3%), reclassifying patients with *possible* IE to either of the two other groups (*definite* or *rejected* IE) [34].TAVI-related IE poses particular challenge to the sensitivity of the modified Duke criteria. The decreased sensitivity of echocardiography is attributed to the location of vegetation and the acoustic shadow of the valve stent [35, 36]. ^18^F-FDG PET/CT may contribute to improved diagnosis [34, 37]. In a small cohort of 16 cases with suspected TAVI-IE, echocardiography showed findings compatible with endocarditis according to the modified Duke criteria in only five out of 10 definite IE cases, while ^18^F-FDG PET/CT was positive for IE in nine cases [37]. In another retrospective multicenter study that evaluated the change in diagnosis with adding ^18^F-FDG PET/CT and/or cardiac CTA in 30 patients with suspected TAVI-IE, the diagnosis had been changed (for both rejection and confirmation) in one-third of the patients [34]. The duration of “physiological” uptake following TAVI and the prognostic value of ^18^F-FDG PET/CT in this setting need further investigation in larger studies.

### Cardiac Implanted Electronic Device Infection

In recent decades, there has been a significant increase in the use of cardiac implanted electronic devices (CIEDs) in clinical cardiology. A sequalae of this has been a rise in CIED infection rates, with diagnosis of this remaining challenging with TTE. Importantly, similar to NVE and PVE, ^18^F-FDG PET/CT is also useful to identify septic emboli originating from cardiovascular devices [27].

#### Diagnosis of Pocket Infection and Lead Involvement

**Fig. 2:**
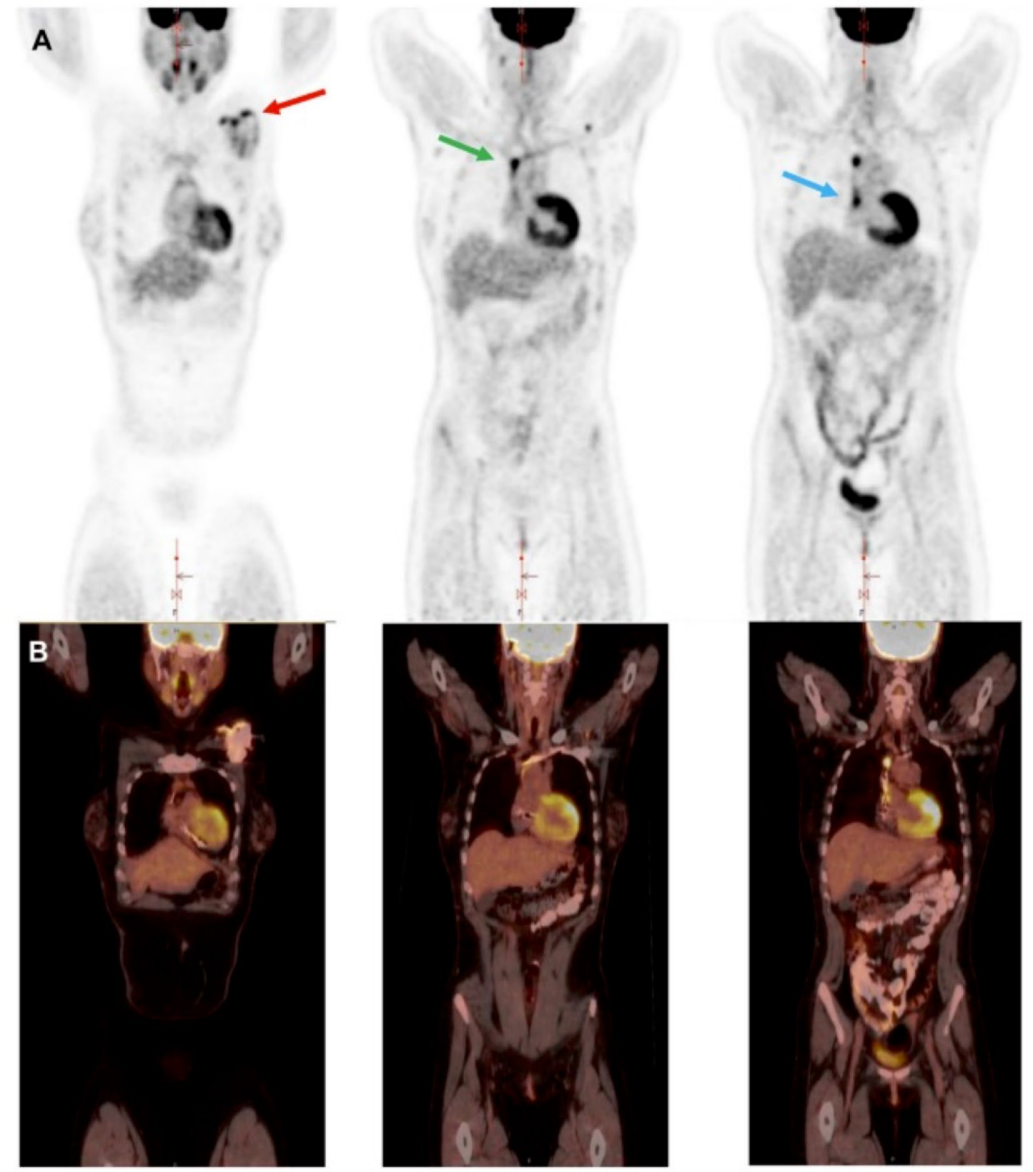
CIED pocket and lead infection diagnosed by ^18^F-FDG PET/CT. Coronal PET images (panel **A**) show multiple areas of focal, heterogeneous, intense ^18^F-FDG uptake surrounding the ICD generator (standardized uptake value, SUV_max_ 4.8, *red arrow*), along the ICD leads (SUV_max_ 6, *green arrow*) and associated with the ICD leads in the right atrium (SUV_max_ 5.9, *blue arrow*). Uptake persisted on non-attenuation corrected images (not shown). Fusion with CT (panel **B**) confirm the anatomic localization. These findings confirmed CIED deep pocket infection with lead involvement. (Adapted from Bourque et al. Journal of Nuclear Cardiology. 2024(34):101786, with permission from Elsevier) [6]

Several studies have specifically investigated the performances of ^18^F-FDG PET/CT for the diagnostic of CIED infection [38–41]. Two recent meta-analyses reported respective sensitivity 83% and specificity 89%, and sensitivity 87% and specificity 94% [8, 38]. Although ^18^F-FDG PET/CT consistently increases the diagnostic accuracy of the modified Duke-Li criteria, its overall sensitivity remains low in suspected CIED infection [18, 42]. A distinction must be made between infection of the extracardiac components of the device (pocket, extracardiac portion of the leads) and involvement of the intracardiac portion of the leads (Fig. 2) [19]. Comparing the performances of ^18^F-FDG PET/CT in these two settings, Jeronimo et al. reported a sensitivity 72.2% and specificity 95.6% for the diagnosis of pocket infection vs. sensitivity 38.5% and specificity 98.0% for lead infection, despite adequate myocardial suppression in both groups [43]. A meta-analysis of 14 studies found a sensitivity of 96% and specificity of 97% for pocket infection versus a sensitivity of 76% and specificity of 83% for lead involvement [38]. Combining ^18^F-FDG PET with CTA reclassifies more patients initially deemed *possible* IE and detects more patterns of IE than nonenhanced CT [12, 44].

## Extracardiac Complications

As noted for valvular IE, the ability to scan the entire body with ^18^F-FDG PET/CT is useful for detection of embolic foci and metastatic sites of infection such as spine infections, mycotic aneurysms, and splenic and pulmonary emboli [45]. These findings can support the diagnosis of lead or valvular endocarditis, as well as help guide appropriate antimicrobial therapy and need for device explantation [12]. Several studies performed in patients with CIED infection have demonstrated that ^18^F-FDG PET/CT could reveal unknown extracardiac infectious foci in about one out of five patients [12, 41].

## Prognosis and Management

The prognostic value of ^18^F-FDG PET/CT in CIED-IE is less documented. A recent study found that patients with confirmed lead CIED-IE but *without*^18^F-FDG uptake around the pocket nor clinical signs of pocket infection (“cold close pocket” infection) experienced worse outcome following lead extraction [46]. If confirmed, these results might indicate a prognostic role for ^18^F-FDG PET/CT prior to lead extraction.

### LVAD Infection

Left ventricular assistant devices (LVAD) are a circulatory support therapeutic option for end-stage heart failure, either as a bridge to heart transplantation or increasingly as destination therapy. LVADs include a pump with inflow and outflow cannulas and a driveline, any part of which can become infected. Device infection can occur in about one out of five patients with LVAD [47] and is associated with high morbidity and mortality [48]. Diagnoses of LVAD infection is particularly challenging, as many components are not evaluated by echocardiography; ^18^F-FDG PET/CT may be helpful in this setting [49]. A recent meta-analysis with pooled results from 8 studies and 256 exams by Ten Hove et al. [50] found sensitivity and specificity of 95% and 91% for the diagnosis of either pocket and/or driveline infection, respectively. Similarly high performances were reported by Tam et al. [51]. ^18^F-FDG PET/CT stigmata of LVAD infection are associated with an increased mortality, in particular in case of central infection [52]. Interestingly, using the metabolic volume performs better than SUV_max_ and visual grading for the diagnosis of LVAD-IE [53].

## Cardiovascular Inflammation

^18^F-FDG PET/CT imaging plays a key role in the diagnosis and management of patients with cardiovascular inflammatory disorders, including sarcoidosis. In addition, there is mounting evidence of increased cardiovascular (CV) morbidity and mortality from autoimmune-mediated rheumatic and musculoskeletal diseases [54]. Although the specific mechanisms underlying each disease and their respective contributions to CV manifestations vary, a common etiologic pathway centers on systemic inflammation [54]. Understanding the CV impact of immune-mediated inflammatory diseases has significant implications for early detection, risk stratification, prognostication, and implementing strategies to mitigate cardiometabolic risk and institute disease-modifying therapies that may alter clinical course [54]. Indeed, nuclear cardiology imaging with ^18^F-FDG PET/CT plays a crucial role in assessing the extent of cardiac involvement across the field of cardio-rheumatology by allowing for a more accurate diagnosis, phenotypic characterization, and monitoring of cardiac diseases [54]. We will focus on cardiac sarcoidosis and large-vessel vasculitis, though there is emerging use and data in other inflammatory disorders.

### Cardiac Sarcoidosis

Active inflammatory cells in sarcoid granulomas avidly take up glucose and its analogs and scar can be identified by perfusion imaging. Thus ^18^F-FDG PET/CT can provide a comprehensive assessment in suspected cardiac sarcoidosis (CS), particularly when combined with whole-body imaging to uncover extracardiac involvement [55]. PET is typically combined with chest CT for co-localization and attenuation correction. As in infection imaging, physiologic cardiac glucose metabolism is switched off by a low-carbohydrate/high-fat diet followed by fasting, and the complex regimen results in a 10–15% diagnostic failure rate [56]. Assessment of LV scar or reversible microcirculatory impairment is obtained from parallel scanning with either PET or single-photon emission computed tomography myocardial perfusion imaging [57]. Abnormal cardiac PET is a key criterion in the diagnostic evaluation of CS [58].

#### Diagnosis

Radionuclide imaging is incorporated in the diagnostic algorithms from the Japanese Circulation Society (JCS) in 2017 [59] and the 2014 Heart Rhythm Society (HRS) expert consensus statement on cardiac sarcoidosis [58]. In the JCS criteria, one of the major clinical criteria is abnormal myocardial uptake of ^18^F-FDG. In the HRS criteria, if a patient has histologically confirmed extracardiac sarcoidosis, then cardiac involvement is probable (among other non-radionuclide imaging-based criteria) if myocardial ^18^F-FDG uptake is present on PET/CT in a pattern consistent with cardiac sarcoidosis. Radionuclide imaging findings suggestive of cardiac sarcoidosis include multiple noncontiguous perfusion abnormalities without associated ^18^F-FDG uptake, focal or focal on diffuse ^18^F-FDG uptake associated with a resting perfusion abnormality, multiple noncontiguous perfusion defects with associated ^18^F-FDG uptake (perfusion/metabolism mismatch), or multiple areas of focal ^18^F-FDG uptake and the presence of extracardiac ^18^F-FDG uptake [60, 61].

In a meta-analysis of 17 studies involving 891 patients with suspected CS, the sensitivity and specificity of PET were 84% and 83%, respectively [62]. The utility may even be better than this as the reference standard is diagnostic criteria, which is imperfect [63, 64]. Absence of extracardiac uptake decreases the specificity of PET for CS [61]. In addition to a visual review of PET images, quantification of inflammation is possible. Several metrics assess intensity and heterogeneity of ^18^F-FDG uptake or myocardial metabolic volume and activity [65, 66] and can provide added value for diagnosis and monitoring of disease progression and response to therapy.

Recently, the complementary role of ^18^F-FDG PET and cardiac MRI in the evaluation of CS has been highlighted in multiple studies [67] and the feasibility of hybrid simultaneous imaging by PET/MR system has emerged [68]. The higher sensitivity of PET/MR over PET and CMR alone is now established [69]. In addition to offering complementary diagnostic utility, ^18^F-FDG PET/CT is particularly useful if cardiac MRI is suggestive of cardiac sarcoidosis to assess the presence of baseline myocardial inflammation and potentially identify ^18^F-FDG-avid extracardiac tissue that may be amenable to biopsy.

#### Prognosis

Apart from its significant role in the diagnosis of cardiac sarcoidosis, radionuclide imaging results impact prognosis in patients with suspected and known cardiac sarcoidosis [70]. For example, in patients with suspected cardiac sarcoidosis not on immunosuppression who were referred for ^18^F-FDG PET/CT, the summed rest score, a surrogate of scar, was associated with adverse outcomes [71, 72].In another report of suspected cardiac sarcoidosis patients, the incidence of all-cause mortality or sustained ventricular tachycardia was 7.3%, 18.4%, and 31.9% for patients with normal myocardial perfusion and no myocardial ^18^F-FDG uptake, abnormal myocardial perfusion or focal myocardial ^18^F-FDG uptake, and abnormal myocardial perfusion and focal myocardial ^18^F-FDG uptake, respectively [64]. Furthermore, abnormal myocardial perfusion and focal ^18^F-FDG uptake was the strongest predictor of the composite outcome of all-cause mortality or sustained ventricular tachycardia. In addition, in suspected cardiac sarcoidosis patients undergoing hybrid ^18^F-FDG PET imaging and cardiac MRI, right ventricular ^18^F-FDG uptake and LGE on cardiac MRI were independent predictors of arrhythmias, hospitalization, or all-cause mortality [69].

## Disease Monitoring and Treatment Effectiveness

**Fig. 3:**
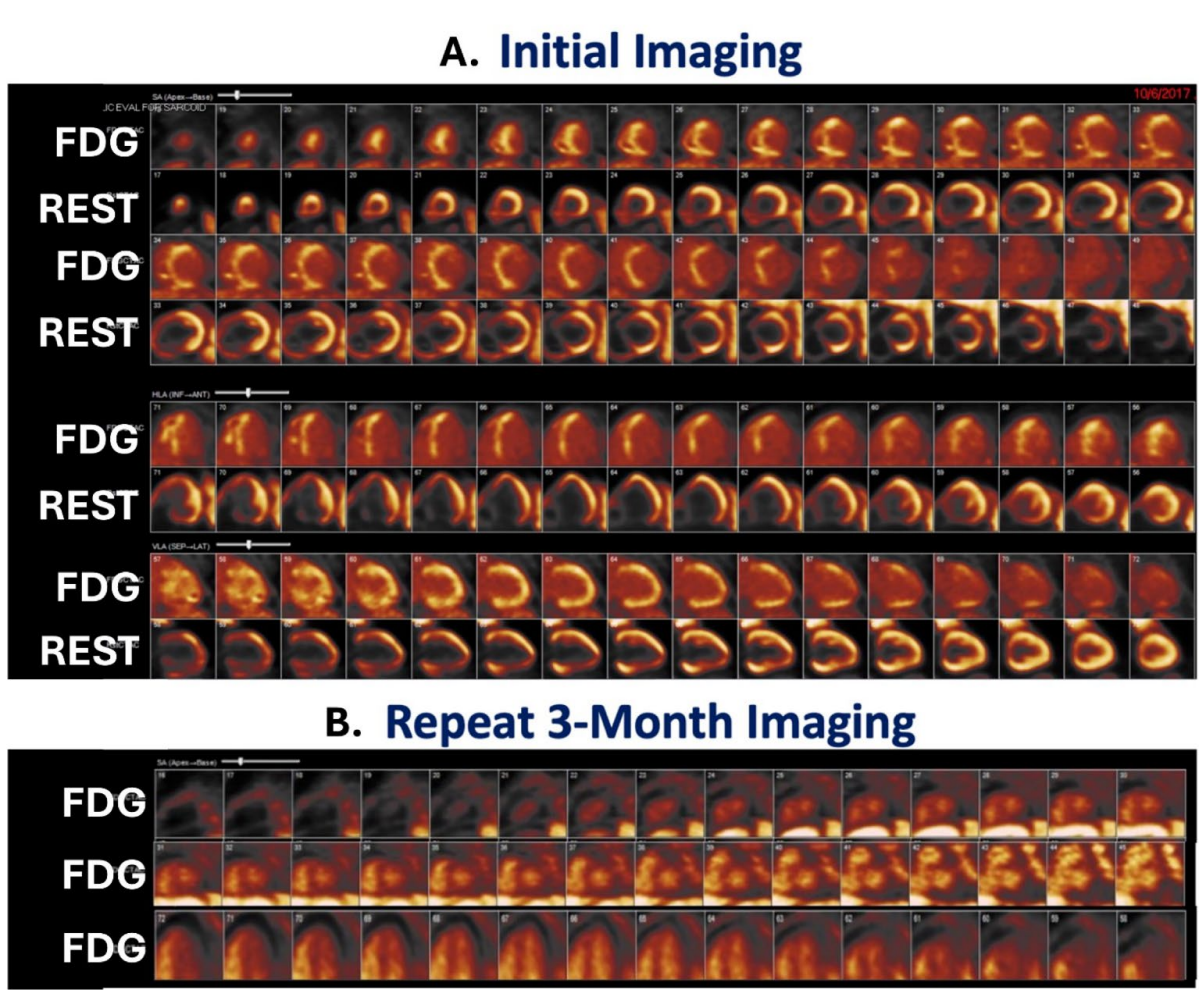
Serial ^18^F-FDG PET/CT imaging to diagnose cardiac sarcoidosis and assess response to therapy. A 67-year-old female with a history of pulmonary sarcoidosis presents with an episode of paroxysmal ventricular tachycardia after three weeks of dyspnea on exertion and orthopnea. She underwent positron emission tomography/computed tomography with ^18^F-fluorodeoxyglucose (FDG) to assess inflammation and ^13^N-ammonia to evaluate perfusion (**A**). Imaging revealed a high concern for active inflammation from cardiac sarcoidosis with prominent heterogeneous ^18^F-FDG uptake in the apical to basal anteroseptum, inferoseptum, and inferior walls and associated perfusion defect in the same territory. She was started on prednisone and transitioned to mycophenolate mofetil and heart failure goal-directed medical therapy. She had resolution of her symptoms. Repeat imaging at 3 months showed complete resolution of her ^18^F-FDG uptake consistent with disease remission (**B**). She was able to taper off therapy successfully

Another important utility of ^18^F-FDG PET/CT in cardiac sarcoidosis lies in the evaluation of disease progression and assessment of immunosuppression over time (Fig. 3) [70, 73]. Emerging data show that serial imaging with ^18^F-FDG PET/CT can help assess alterations in visual assessment of perfusion/metabolism, quantified intensity and volume of myocardial inflammation, LV systolic function, and extracardiac inflammation [74, 75]. For example, there was a significant inverse linear relationship between myocardial SUV _max_ and LVEF and volume of inflamed myocardium and LVEF in 23 cardiac sarcoidosis patients followed serially over two years [76]. In another study, lack of improvement in metabolic activity on serial ^18^F-FDG PET/CT studies was the only variable associated with the composite prognostic endpoint in 20 cardiac sarcoidosis patients undergoing ventricular tachycardia ablation [56, 77]. There are other small studies with similar results.

### Large-Vessel Vasculitis

#### Clinical Application

Large-vessel vasculitis (LVV) involves inflammation of the aorta and its main branches, with giant cell arteritis (GCA) and Takayasu’s arteritis (TAK) as the 2 major forms [78]. There is a substantial incidence, particularly among those of Northern European descent [79]. Clinical assessment of vascular inflammation in the aorta and its primary branches is critically important for diagnosis and prognosis but is challenging due to inconsistent clinical symptoms [80]. Symptoms often indicate damage to the large arteries with compromise of the vascular lumen. Once a patient experiences significant luminal damage, the therapeutic window to preserve undamaged vasculature may have been missed. Accurately monitoring these diseases over time can be problematic, as symptoms are variable and acute phase reactants aren’t specific. Catheter-based fluoroscopic angiography and non-invasive angiography has been used to visualize vascular damage in LVV. Societies have recently endorsed expansion of the vascular imaging toolbox for the clinical management of LVV. The 2021 American College of Radiology Appropriateness Criteria for noncerebral vasculitis designated ^18^F-FDG PET imaging as “usually appropriate” [81]. The 2018 European Alliance for Associations of Rheumatology (EULAR) recommends that LVV should be confirmed using biopsy or imaging including use of ^18^F-FDG PET/CT [82].

#### Diagnosis

**Fig. 4:**
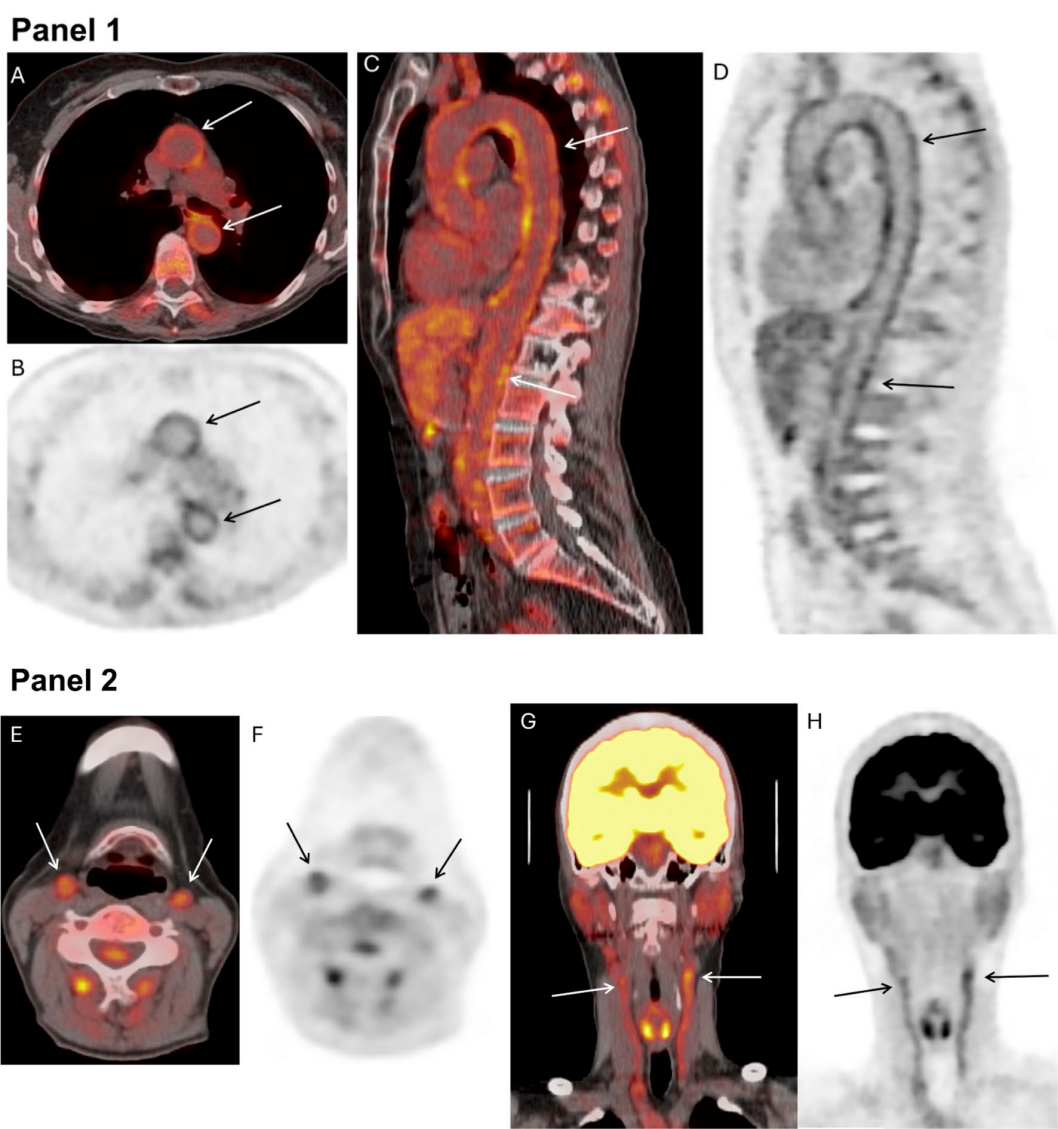
Active large vessel vasculitis. A 63-year-old female with a recent diagnosis of giant cell arteritis had an ^18^F-fluorodeoxyglucose (^18^F-FDG) PET/CT performed to assess for extent/activity of disease elsewhere. A Vasculitis FDG PET/CT was performed from vertex of the skull through the mid thighs, with a dedicated head and neck bed acquisition. Panel 1: Fusion and PET images are displayed, images **A** and **B** correspond to the axial plane at the level of ascending and descending aorta, while images **C** and **D** are oriented to the thoracoabdominal aorta. There is concentric FDG uptake along all the segments of the aorta (Images **A-D**, *white arrows* on fusion images and *black arrows* on PET images), consistent with active vasculitis. Panel 2: Fusion and PET images of the head and neck are displayed, images **E** and **F** correspond to the axial plane at the level of common carotid arteries, while images **G** and **H** correspond to the coronal plane. There is abnormal FDG uptake along the common carotid arteries (Images **E**-**H**, *white arrows* on fusion images and *black arrows* on PET images), consistent with active vasculitis of the common carotid arteries in the context of known giant cell arteritis

Three clinical scenarios are commonly encountered when considering the use of ^18^F-FDG PET/CT imaging to diagnose LVV (Fig. 4). In patients with suggestive symptoms of LVV, ^18^F-FDG PET/CT imaging can be used to confirm the diagnosis with excellent performance characteristics. A meta-analysis reported a pooled sensitivity of 76% and specificity of 93% [83] for this indication. Glucocorticoid use may reduce the sensitivity of ^18^F-FDG PET/CT imaging [84]. ^18^F-FDG PET/CT can also be used in fever or inflammation of unknown origin. A large proportion of patients who present with non-specific constitutional symptoms including unexplained fever may eventually be diagnosed with LVV [85]. However, interpretation of borderline abnormalities on vascular PET in this context must be performed with caution, and should be supported with complimentary imaging studies or temporary artery biopsy. Finally, in patients with structural abnormalities of large arteries incidentally noted on non-invasive imaging, particularly in oncology patients using checkpoint inhibitors or granulocyte-colony stimulating factors, use of ^18^F-FDG PET/CT may help to define these abnormalities further [86].

#### Disease Monitoring

Although ^18^F-FDG PET/CT image can play an important role in LVV diagnosis, the use of serial PET imaging to monitor disease progression and response to therapy in this condition is not clearly defined [80]. On the one hand, ^18^F-FDG PET/CT may detect vascular inflammation and allow treatment decisions prior to irreversible vascular damage; on the other hand, a significant number of LVV patients in cross-sectional studies have evidence of active vasculitis on ^18^F-FDG PET/CT imaging during apparent clinical remission [87]. There are very few prospective observational studies that have addressed this topic, and it is unclear whether therapy should be altered in patients primarily based on subclinical imaging findings [80, 88].

There is emerging data suggesting that ^18^F-FDG PET/CT imaging may facilitate monitoring of treatment response [80]. Many observational studies have used PET vascular activity score (PETVAS), a summary score of ^18^F-FDG uptake throughout the large arteries, as an outcome measure of vascular inflammation [89, 90]. Whether ^18^F-FDG PET/CT imaging guides prognosis is unclear.

#### Novel Radiotracers

A possible challenge to the clinical application of ^18^F-FDG PET/CT in vascular imaging is the lack of specificity [80]. This is because arterial wall glycolysis may be due to inflammation from invading immune cells, vascular remodelling/repair, or other pathology such as secondary atherosclerosis [80]. Emerging radiotracers that focus specifically on immune subsets may fill the void and provide an in vivo evaluation surrogate to histologic assessment [80]. This is because LVV pathogenesis is partly due to immune cell subsets [79]; macrophages and activated T helper cell subsets are involved in both TAK and GCA [80]. A number of radiotracers apart from ^18^F-FDG have been put forward and are currently being tested for various indications [91, 92]. The main issues relate to non-specific blood pool activity in radiotracers that work on hematopoietic cells, and identifying vasculitis from other pathologies may still be a problem even with immune-specific radiotracers [80]. Clinical application in LVV remains to be determined.

## Conclusions

Radionuclide imaging with ^18^F-FDG PET/CT is an essential component in the armamentarium of cardiovascular imaging techniques for the assessment and management of many complex cardiac pathologies, including infectious and inflammatory diseases. This review has highlighted its critical role in valvular infective endocarditis, known and suspected CIED infection, LVAD infections, cardiac sarcoidosis, and large vessel vasculitis.

## Data Availability

No datasets were generated or analysed during the current study.
